# Toxicokinetics of Nanoparticles Deposited in Lungs Using Occupational Exposure Scenarios

**DOI:** 10.3389/fpubh.2022.909247

**Published:** 2022-06-21

**Authors:** Otto Creutzenberg, Gerhard Pohlmann, Dirk Schaudien, Heiko Kock

**Affiliations:** Fraunhofer Institute for Toxicology and Experimental Medicine, Hanover, Germany

**Keywords:** nanoparticles, agglomerates, toxicokinetics, translocation, metal oxides, carbon black

## Abstract

Various synthetic powders with primary particle sizes at the nanoscale and a high commercial impact have been studied using Wistar rats. The test materials were metal oxides, i.e., TiO_2_, ZnO and amorphous silica, and carbon black (technical soot). Dosing schemes were in the regular ranges typically used in subacute rat studies to simulate occupational exposure scenarios (mg range). Nanoscaled particle agglomerates have the potential to disintegrate and translocate as individual nanoparticles to remote locations following deposition in the lungs. The toxicokinetic fate of metal oxides post-inhalation in lungs/organs was investigated (i) by chemical analysis of the retained particulate/dissolved matter and (ii) by visualization of particles in various remote organs using transmission electron microscopy (TEM). The three titanium dioxides (NM-103, NM-104, NM-105; JRC coding) showed a very slow dissolution in lung fluids. In contrast, the coated ZnO (NM-111) dissolved quickly and was eliminated from the body within approximately 1 day. The precipitated amorphous silica (NM-200) showed a partial dissolution. Chemical analysis in lungs (particulate and soluble TiO_2_) and in remote organs (liver and brain) showed a small solubility effect under physiological conditions. The translocation to remote organs was negligible. This confirms that for poorly soluble TiO_2_ particles there was no considerable translocation to the liver and brain. The chemical analysis of zinc demonstrated a very rapid dissolution of ZnO particles after deposition in the lungs. Statistically significant increases in Zn levels in the lungs were detectable only on day 1 post-exposure (NM-111). Overall, no relevant amounts of increased NM-111 in the ionic or particulate matter were detected in any body compartment. Amorphous silica (NM-200) particles were found in the cytoplasm of intraalveolar macrophages in the lung and the cytoplasm of macrophages in the lung associated lymph node. Interestingly, these particles were found in a few animals of all treatment groups (1, 2.5, and 5 mg/m^3^ NM-200) even after 91 days post-exposure. In all other organs of the NM-200 treated animals such as the nasal epithelium, trachea, larynx, liver, spleen, kidney, and mesenteric lymph node no particles were found at any time point investigated. Carbon black was tagged internally (“intrinsically”) with a γ tracer (^7^beryllium; half-time: 53.3 days). Due to limited amounts, the test item (0.3 mg per rat lung) was intratracheally instilled into the lungs. This dose avoided a particle overload effect, meaning that the toxicokinetic fate of carbon black could be followed under the approximated physiological conditions of lung clearance. Analysis of the γ labeled carbon black confirmed conclusively that there was no evidence for the translocation of carbon black beyond the lung into the blood or other body compartments. Very small amounts were only detected in lung-associated lymph nodes (LALN). On day 20 post-treatment, upon necropsy, both carbon black samples were practically exclusively found in lungs (75.1% and 91.0%, respectively) and in very small amounts in the lung-associated lymph nodes (LALN), i.e., ~0.5%. In the other organs/tissues, the test item was not significantly detectable. Separation of leukocytes and cell-free supernatant of a bronchoalveolar lavagate by centrifugation revealed that carbon black was completely located in the cell sediment, indicating total engulfment by alveolar macrophages. In conclusion, in occupational settings the nanomaterials titanium dioxide, zinc oxide, amorphous silica, and carbon black acted as microscaled agglomerates, not as individual nanoparticles. They displayed no potential to translocate beyond the lung into the blood compartment. Besides lungs, very small particulate amounts were detected only in LALN. This finding is consistent with the behavior of microscaled poorly soluble particles. Overall, there was no evidence of translocation of the nanomaterials following pulmonary exposures.

## Introduction

The toxicokinetics of inhaled nanoparticles is predominantly determined by **deposition** characteristics (efficiency, aerodynamic particle size), **dissolution** behavior (in biological media), and **translocation** potential (driven by size-depending migration pathways). In this context, individual nanoparticles behave differently to agglomerated ones with regard to their fate following deposition in the lungs. Various papers have reported biokinetic inhalation studies using extremely small aerosol concentrations ( ≤ 1 μg/m^3^) and highly sensitive analytical methods (1, 2). Occupational concentrations of poorly soluble particles are in the range of ~1 mg/m^3^ while environmental levels may reach up to 50 μg/m^3^ (3). In the case of metal oxides, such aerosols consisting of individual nanoparticles can be experimentally established with spark generators and analytics can be refined using the isotope technique. Risk assessment of occupational exposure scenarios in rodent studies uses aerosol concentrations in the range of 1–60 mg/m^3^. Dry dispersion of the test powders results in microscaled mean particle sizes even though the test item may have primary particle sizes in the nano range. The reason for this is that at the given aerosol concentrations agglomerate formation is necessarily the dominating implication (4–6). In this perspective article, the toxicokinetic outcome of studies conducted for risk assessment in working areas is discussed. Examples of various studies on **nano-TiO**_**2**_**, nano-ZnO**, and **nano-SiO**_**2**_ as well as **carbon black** were investigated.

## Materials and Methods

### Metal Oxides

#### Exposure Conditions

The three test items were taken from the repository of the Joint Research Centre, Ispra, Italy, and aerosolized using a dry dispersion technique. The dispersion was achieved by a feeding system and a high-velocity pressurized air dispersion nozzle developed by Fraunhofer ITEM (7). Concentrations were recorded using an aerosol photometer (scattering light signal). Wistar rats were used as a test model and were exposed for 6 h/day, 5 days/week. The aerosols were generated by a flow-past nose-only inhalation exposure system.

#### Titanium Dioxides

A 28-day nose-only inhalation toxicity study in rats was performed with three TiO_2_ varieties: NM-103 (primary particle diameter: 20 nm/rutile/silicone-coated/hydrophobic), NM-104 (PPD: 20 nm/rutile/glycerol-coated/hydrophilic), NM-105 (PPD: 22 nm/anatase-rutile 80%-20%/untreated/hydrophilic). The aerosol concentrations selected were 3, 12, and 48 mg/m^3^ for each test item and simulated conditions at an occupational exposure scenario converted to a typical dosing scheme of a subacute rat study. In addition to the regular study design endpoints (OECD 412), investigations on the disintegration of agglomerates in lungs and the identification of the respiratory cell types responsible for uptake (by TEM analysis) were included. Chemical analysis of the retained masses in the lungs was performed on days 1, 28, and 90 post-exposure.

#### Zinc Oxide

A 90-day nose-only inhalation toxicity study in rats was performed with a coated nanoscaled zinc oxide sample (NM-111; coated with triethoxycapryl silane). The aerosol concentrations selected were 0.3, 1.5, and 4.5 mg/m^3^. In addition to the regular study design endpoints (OECD 412), TEM investigations to detect ZnO particles in lungs were performed (magnification up to 40,000 × to allow detection of primary nanoparticles). A chemical analysis of the retained masses in the lungs was performed on days 1 and 30 post-exposure (total of Zn^2+^ and ZnO particles).

#### Amorphous Silica

A 90-day nose-only inhalation toxicity study in rats was performed with an amorphous silica sample (NM-200; synthetic amorphous silica, precipitated). The aerosol concentrations selected were 1, 2.5, and 5 mg/m^3^. TEM investigations were performed to detect SiO_2_ particles in lungs (magnification up to 40,000×); two samples of the lung and one sample of trachea, larynx, nasal epithelium, lung associated lymph node, liver, spleen, kidney, and mesenteric lymph node were investigated of all animals of the treatment groups and of all time points (1, 30, and 90 days post exposure); control group rats served to acquire the normal biological background of electron dense structures. To achieve better visibility of possible particles in comparison to the biological background, ultrathin sections were not contrasted using uranyl acetate and lead citrate. Chemical analysis of the retained masses in the lungs was performed on days 1, 30, and 90 post-exposure.

### Carbon Black

#### Test Items

Two carbon black samples i.) Monarch^®^ 1,000 (oxidized carbon black grade) from Cabot Corp., USA. and ii.) Printex^®^ 90 (untreated carbon black grade)] from Orion Engineered Carbons GmbH, Germany were investigated. Carbon black is a black, finely divided powder, consisting of aggregates (in the size range between 100 and 1,000 nm) of aciniform morphology (i.e., aggregates that have been strongly fused in a random configuration that resembled grape-like clusters. The carbon black aggregates rapidly form larger agglomerates held together by van der Waals forces.

Production of ^7^Be-tagged carbon black: Technical soot (carbon black) is synthesized by a thermal process initially forming nanoscaled primary particles. Due to aging the material finally builds aggregates in the size range of 100–1,000 nm and the latter stick together to form microscaled agglomerates.^7^Be-tagged carbon black can be produced by direct irradiation of carbon black (8, 9). Using the proton irradiation technique the ^7^Be radioisotope is produced directly in the crystal lattice of carbon without evidently altering the material structure. Nuclear reaction: natC(p,x)^7^Be, mainly *via* ^12^C(p,3p3n)^7^Be channels; proton energy range 24–38 MeV; ZAG Zyklotron, Karlsruhe, Germany; ^7^Be is a γ tracer with a half-time = 53 days. As only ~20 mg of carbon black can be activated per run the test item was sufficiently available for an intratracheal instillation study only; however, this approach is a good surrogate. In this study two carbon black samples [Monarch^®^ 1,000 (oxidized carbon black grade) and Printex^®^ 90 (untreated carbon black grade)] were labeled with ^7^Be.

Purification of the ^7^Be-tagged carbon black: The test item was liberated from soluble or loosely attached moieties using a triplicate of solvents (EtOH/H_2_O 1/1 v/v−0.01 N HCl—Artificial lysosomal fluid—ALF) to consecutively wash and filter the carbon black samples (10). An aqueous suspension of ~0.3 mg of the ^7^Be-carbon black sample was taken in a syringe and the carbon black was separated by pressing through a filter (nuclear pore size: 0.4 μm).

#### Administration of the ^7^Be-Tagged Carbon Black Samples

Test items were intratracheally instilled into the lungs. A single dose of ~0.3 mg carbon black suspended in 0.3 ml of sterile isotonic saline was administered. This dose avoided a particle overload effect, thus, the toxicokinetic fate of the test item could be followed under approximated physiological conditions of lung clearance (note that bolus effects could occur following intratracheal instillation).

## Results

### Metal Oxides

#### Titanium Dioxides

The chemical analysis of test items retained in the lungs matched the values predicted by the Multipath Particle Dosimetry (MPPD) model v. 3.04 (11). On day 3 post-exposure, in the low dose groups 0.4, 0.4, and 0.5 mg/lung, in the mid dose groups 1.6, 1.7, and 1.8 mg/lung and in the high dose groups 7.0, 3.8, and 5.9 mg/lung were determined for NM-103, NM-104, and NM-105, respectively. The retained masses showed a high similarity in the groups of the same dose. From the calculated retention half-times (kinetic data of the 3 time-points on days 3, 45, and 90 post-exposure) a non-overload, a slight overload, and a moderate overload could be deduced for the low, mid, and high dose groups (Table 1).

**Table 1:** Retention half-times of test items in lungs.

**Retention half-times in lungs (days)**	**NM-103**	**NM-104**	**NM-105**
Low dose	59	85	46
Mid dose	162	267	204
High dose	373	315	485

*For comparison: Half-time untreated rat is ~60 days*.

During the recovery period, almost no lung clearance was observed in the high dose groups. In contrast, in the mid and low dose groups, a partial and a physiological lung clearance were found, respectively. This reflects the different grades of clearance retardation due to the various lung loads.

The soluble moiety of the test items in the lungs - as measured by separating the particulate and dissolved parts by filtration (0.2 μm pore diameter) - reached up to 5.5% of the total mass in the low dose groups; however, it was not more than 2.2 and 0.9% in the mid and high dose groups, respectively. These results suggest that the solubility of the test item is limited by a given maximum under the conditions of the lung ambiance.

The translocation potential from the lungs was very small. TEM analysis revealed that intraalveolar macrophages are the most prominent compartment of particle detection. In the ranking, the compartment pneumocytes type I cells (low and mid doses) and free particles (high doses) finished second.

#### Zinc Oxide

One day after the end of exposure, in the high dose group of NM-111 the absolute Zn content (35 μg/lung) was slightly increased (statistically significant) to 180% in the lung as compared to the clean air control group. In all other organs and also at day 30 post-exposure the Zn levels were very close to the control values.

#### Amorphous Silica

Lung burdens of 91, 172, and 307 μg/lung were analyzed on day 1 after the end of exposure in the low, mid, and high dose groups, respectively. Data at 1 and 3 months post exposure revealed that, in addition to the ~60 day half-time of physiological clearance, a dissolution effect is the reason for the calculated actual half-times of 30, 32, and 28 days respectively in the low, mid and high dose groups. The chemical analysis of silicon confirmed an evident dissolution of NM-200 under lung ambient conditions. Statistically significant increases in silicon levels in the lungs were detectable on days 1, 30, and 90 post-exposure (NM-200). TEM analysis confirmed the presence of SiO_2_ particles in lungs/LALN up to day 90 post-exposure. In these spot checks, in the NM-200 high dose group SiO_2_ particles (confirmation by EDX) were detected within the cytoplasm of intraalveolar macrophages (TEM analysis). However, those particles were not detectable in remote organs (nasal epithelium, trachea, larynx, liver, spleen, kidney, and mesenteric lymph node).

### Carbon Black

#### Feces/Urine

**Figure 1:**
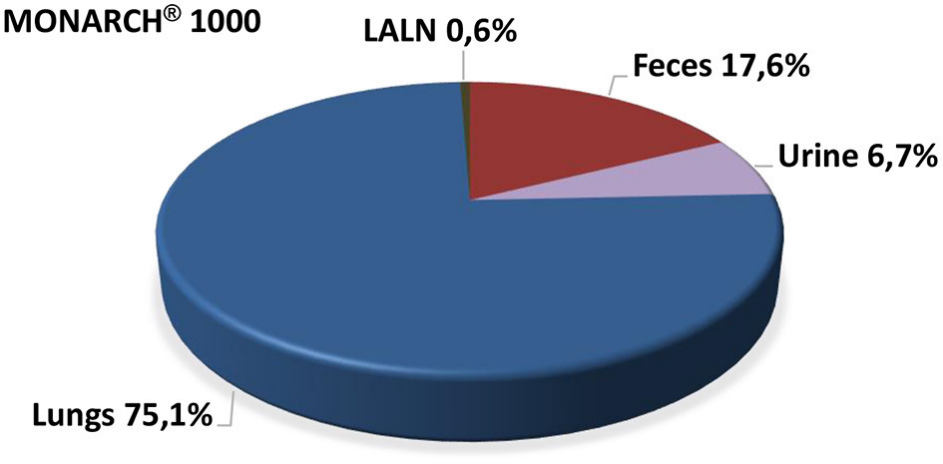
Percentage of body distribution of MONARCH^®^ 1000 post instillation (the entire radioactivity detected in the rat body was set as 100%).

**Figure 2:**
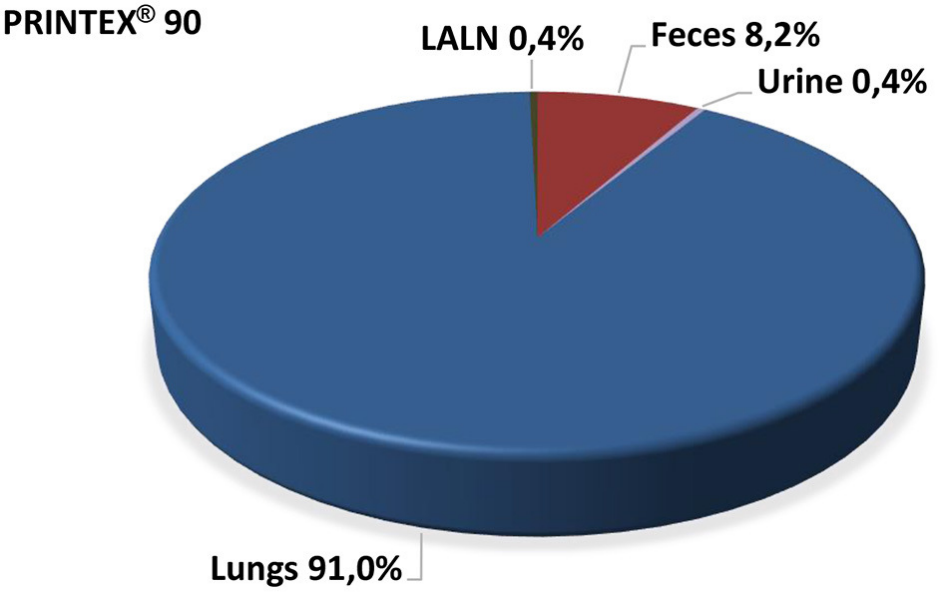
Percentage of body distribution of PRINTEX^®^ 90 post instillation (the entire radioactivity detected in the rat body was set as 100%).

In feces and urine, up to day 3 post-treatment ^7^Be-Monarch^®^ 1,000 was detectable and reached mean values of 17.6 and 6.7%, respectively (Figure 1). For ^7^Be-Printex^®^ 90 the corresponding data were lower and reached 8.2 and 0.4% (Figure 2). The percentages measured in feces may have been swallowed directly after administration or after alveolar macrophage clearance. As no significant amounts of the two grades were detected in blood, the levels measured in urine can be traced back to cross-contamination during the feces/urine collection in the metabolic cages.

#### Blood Kinetics

In blood samples, the two ^7^Be-carbon black grades were not detectable at significant levels (up to samples collected on days 1–3). Consequently, a maximum concentration (c_max_) of the test items in the blood (where the organs could have been determined most precisely) could not be identified.

#### Organs/Tissues

All organs and tissues were analyzed on day 20 post-treatment for radioactivity. ^7^Be-Monarch^®^ 1000 was detected only in the lungs as the deposition site (75.1%) and, at a low level (0.6%), in the lung-associated lymph nodes (LALN) (Figure 1). Correspondingly, ^7^Be-Printex^®^ 90 values were 91.0 and 0.4% in the lungs and LALN, respectively. In all other organs (including male reproductive organs) no significant amounts of the test items were detected (Figure 2).

#### ^7^Be-Printex^®^ 90 Analysis in Bronchoalveolar Lavage Fluid

Exhaustive lung lavages were performed to harvest a large moiety of the lung leukocyte pool. Subsequent measurements of the radioactivity revealed that ~50% of the total ^7^Be-Printex^®^ 90 retained in lungs had been gained by the lavage process. Centrifugation of the bronchoalveolar lavagate showed that the distribution in cell sediment/cell-free supernatant was 100/0%. Cytoslides of the lung leukocytes showed that the carbon black was fully engulfed by alveolar macrophages.

## Discussion

### Metal Oxides

The three titanium dioxides (NM-103, NM-104, NM-105) are examples of very slowly soluble nanomaterial in lung fluids. In contrast, the coated ZnO (NM-111) shows quick dissolution and is eliminated from the body within ~1 day. The precipitated amorphous silica (NM-200) shows a partial dissolution. The variation of dissolution behavior among different nanoforms has been discussed for grouping approaches by Keller et al. (12).

#### Titanium Dioxides

The chemical analysis in lungs (particulate and soluble TiO_2_) and in remote organs (liver and brain) showed a small solubility effect under physiological conditions. The translocation to remote organs was negligible. This confirms for the poorly soluble TiO_2_ particles to have no considerable translocation to the liver and brain.

#### Zinc Oxide

The chemical analysis of zinc demonstrated a very rapid dissolution of ZnO particles after deposition in the lungs. Statistically significant increases of Zn levels in the lungs were detectable only on day 1 post-exposure (NM-111); increases of zinc were no longer observed on days 14 or 30 post-exposure. ZnO particles in tissues were not detectable by TEM. The deposited mass of NM-111 in the 90-day exposure period was ~2,000 μg/lung (calculated by MPPD model), the analytical results thus demonstrate a practically complete dissolution of the retained test item. Overall, no relevant amounts of increased NM-111 in the ionic or particulate matter were detected in any body compartment.

#### Amorphous Silica

Within the NM-200 treated animals particles were found that consisted of silicon using EDAX-analysis. These particles were found in the cytoplasm of intraalveolar macrophages in the lung and the cytoplasm of macrophages in the lung associated lymph node. Interestingly, these particles were found in a few animals of all treatment groups (1, 2.5, and 5 mg/m^3^ NM-200) even after 91 days post exposure. In all other organs of the NM-200 treated animals, such as the nasal epithelium, trachea, larynx, liver, spleen, kidney, and mesenteric lymph node, no particles were found at any time point investigated. Furthermore, no particles were detected in the investigated organs of the clean air treated animals.

### Carbon Black

The use of a γ tracer tightly bound into the graphite lattice of carbon black enabled proper analysis of the test items in a biological system. The structure of technical soots (carbon black) is characterized by a strong tendency of the aggregates (size range 100–1,000 nm) to form bigger agglomerates at the micrometer scale. The γ tracer analysis confirmed conclusively that there was no evidence for translocation of carbon black beyond the lung into the blood or other body compartments (very small amounts only detected in lung-associated lymph nodes (LALN). After deposition in lungs, carbon black test items act as insoluble agglomerates in μm size. In occupational inhalation exposure scenarios particles fulfilling the nano definition form agglomerates. Their deposition and translocation behavior is the same as observed for microscaled particles. Particles as such do not translocate to remote organs; in most cases, dissolved moieties of the particulate mass deposited in the lungs are the active agent in remote organs.

## Author Contributions

OC: study design, study director of all studies, and experimental conduct of the carbon black studies. GP: aerosol generation. DS: TEM investigation. HK: chemical analyses. All authors contributed to the article and approved the submitted version.

## Funding

The inhalation study with three titanium dioxides was funded by BAuA (German Federal Institute for Occupational Safety and Health)—Project # F 2246. The inhalation studies with zinc oxide and amorphous silica were funded by the European Chemical Industry Council (CEFIC); Project: N1—Tiered approach to testing and assessment of nanomaterial safety to human health. The toxicokinetic study on carbon black was funded by the International Carbon Black Association (ICBA).

## Conflict of Interest

The authors declare that the research was conducted in the absence of any commercial or financial relationships that could be construed as a potential conflict of interest.

## Publisher's Note

All claims expressed in this article are solely those of the authors and do not necessarily represent those of their affiliated organizations, or those of the publisher, the editors and the reviewers. Any product that may be evaluated in this article, or claim that may be made by its manufacturer, is not guaranteed or endorsed by the publisher.
